# Association between *Helicobacter pylori* infection and non-alcoholic fatty liver disease in North Chinese: a cross-sectional study

**DOI:** 10.1038/s41598-019-41371-2

**Published:** 2019-03-19

**Authors:** Tian Jiang, Xia Chen, Chenmei Xia, Huamin Liu, Haifan Yan, Guoping Wang, Zhongbiao Wu

**Affiliations:** 1 Research Center for Translational Medicine, The Affiliated Wenling Hospital of Wenzhou Medical University, Wenling, 317500 Zhejiang, China; 2 Department of Urology, The Affiliated Wenling Hospital of Wenzhou Medical University, Wenling, 317500 Zhejiang, China; 3 Department of Gastroenterology, The Affiliated Wenling Hospital of Wenzhou Medical University, Wenling, 317500 Zhejiang, China; 4 School of Public Health, Taishan Medical University, Tai’an, 271000 China

## Abstract

Non-alcoholic fatty liver disease (NAFLD) is a common liver disease. Previous studies on the association between *Helicobacter pylori* (HP) infection and NAFLD are inconsistent. Our study was aimed to find out the relationship between HP infection and NAFLD. We performed a large cross-sectional study in northern Chinese adults in 2015. ^13^C-urea breath tests were used to determine HP infection status. Abdominal ultrasonography was performed to diagnose NAFLD. Multivariable logistic regression was conducted to identify the association between HP infection and NAFLD. A total of 4081 individuals were included in this study; 2137 (52.36%) participants were HP-positive, and 1022 (47.82%) were diagnosed with NAFLD in HP-positive individuals. The odds ratios (OR) and 95% confidence intervals (CI) of participants with HP infection for NAFLD were 1.20 (1.06–1.36) in crude model and 1.27 (1.07–1.50) in fully adjusted model. When stratified by sex and dyslipidemia, the fully adjusted OR and 95% CI for NAFLD were 1.22 (1.10–1.80) in females and 1.44 (1.18–1.75) in subjects with dyslipidemia. There were not significant increased OR for NAFLD when stratified by age. The study indicate that HP infection is associated with NAFLD, particularly in females and patients with dyslipidemia, suggesting that HP eradication might be an alternative method for the prevention or treatment of NAFLD treatment.

## Introduction

Non-alcoholic fatty liver disease (NAFLD) is a common liver disease that refers to a spectrum of lesions ranging from simple steatosis to steatohepatitis^1^. NAFLD has been recognized as a serious public health issue because it is associated with an increased risk for chronic kidney disease, type 2 diabetes mellitus and cardiovascular disease, as well as a number of other severe chronic diseases^2–4^. The prevalence of NAFLD is approximately 25% in China^5,6^. In addition, the prevalence of NAFLD has increased rapidly in recent years, from 23.48% in 2007 to 44.31% in 2013 for males, and from 17.56% in 2007 to 43.06% in 2013 for females, in northeastern Chinese^7^. Because of the high prevalence, unclear etiology and difficult treatment of NAFLD, finding the risk factors for NAFLD will provide the insights for novel prevention or treatment strategies for NAFLD.

*Helicobacter pylori* (HP) is a Gram-negative and micro-aerophilic bacteria that successfully colonize in the human stomach. HP infection lead to duodenal ulcer disease, gastric ulcer disease, gastric adenocarcinoma, and gastric lymphoma^8^. An association between HP infection and NAFLD would be intriguing because HP infection is present in more than half of the world’s population^9^. Recently, HP infection was thought to be associated with remodeling of gut microbiota^10^. The gut microbiota constitutes an internal environmental factor that drives the progression of NAFLD by influencing whole-body glucose homeostasis and liver lipid metabolism^11,12^. Manipulating the gut microbiota may be a new strategy for preventing or treating NAFLD^13^.

In the past several years, several studies in the association between HP infection and extragastric manifestations have conducted. HP infection is associated with cardiovascular, neurologic, hematologic, head and neck, and uro-gynecologic diseases, as well as diabetes mellitus and metabolic syndrome (MS)^14^. Recently, several studies showed that HP infection may contribute to the pathogenesis of NAFLD, and patients with HP infection may be at an increased risk of NAFLD^15,16^. However, some studies showed that HP infection is not associated with NAFLD^17,18^. Therefore, the role of HP infection in the pathogenesis of NAFLD remains unclear. The previous studies were mostly not population-based and with relatively small sample size^16^. Thus, we conducted a large cross-sectional study in the general population of northern China to explore the association between HP infection and NAFLD.

## Results

### General Characteristics of the Subjects

A total of 4081 individuals were included in this study; 1887 (46.24%) were males, and 2194 (53.76%) were females, with an average age of 44.57 ± 13.56 years. The prevalence of HP was 52.36%, with 1022(47.82%) were diagnosed with NAFLD in HP-positive individuals. The clinical and demographic characteristics are displayed in Table 1. The males have a higher prevalence of NAFLD (*P* < 0.01). BMI, ALT, AST, AKP, UA, and UREA values were higher in the NAFLD group than in the non-NAFLD group (*P* < 0.01). By contrast, AST/ALT levels were lower in the NAFLD group than in the non-NAFLD group (*P* < 0.01). In addition, smoking, education level, hypertension, diabetes, dyslipidemia, MS, and HP infection were significantly different in participants with or without NAFLD (*P* < 0.01).

**Table 1:** Demographic and Clinical Characteristics of the Participants.

Variables	Total	NAFLD		*P*-value
		No	Yes	
(n, %)	4081	2217 (54.32)	1864 (45.68)	
Age (Years)	44.57 ± 13.56	42.37 ± 13.36	47.18 ± 13.33	<0.001
Sex				<0.001
Male (n, %)	1887 (46.24)	774 (41.02)	1113 (58.98)	
Female (n, %)	2194 (53.76)	1443 (65.77)	751 (34.23)	
Education level (n, %)				<0.001
Illiteracy/Primary	256 (6.27)	125 (48.83)	131 (51.17)	
Middle School	1342 (32.88)	642 (47.84)	700 (52.16)	
College/University	2164 (53.03)	1278 (59.06)	886 (40.94)	
Not clear	319 (7.82)	172 (53.92)	147 (46.08)	
Smoking status (n, %)				<0.001
No	3277 (80.30)	1904 (58.10)	1373 (41.90)	
Yes	804 (19.70)	313 (38.93)	491 (61.07)	
Hypertension (n, %)				<0.001
No	2915 (71.43)	1846 (63.33)	1069 (36.67)	
Yes	1166 (28.57)	371 (31.82)	795 (68.18)	
Diabetes (n, %)				<0.001
No	3724 (91.25)	2126 (57.09)	1598 (42.91)	
Yes	357 (8.75)	91 (25.49)	266 (74.51)	
MS (n, %)				<0.001
No	2186 (53.57)	1653 (75.62)	533 (24.38)	
Yes	1895 (46.43)	564 (29.76)	1331 (70.24)	
Dyslipidemia (n, %)				<0.001
No	1396 (34.21)	1099 (74.36)	297 (21.28)	
Yes	2685 (65.79)	1118 (41.64)	1567 (58.36)	
BMI (kg/m^2^)	24.51 ± 4.61	22.59 ± 4.73	26.83 ± 3.18	<0.001
ALT (U/l)	21.40 ± 17.44	16.23 ± 10.53	27.54 ± 21.56	<0.001
AST (U/l)	21.51 ± 10.03	19.82 ± 8.67	23.51 ± 11.12	<0.001
AST/ALT	1.17 (0.89–1.45)	1.33 (1.08–1.60)	0.95 (0.75–1.21)	<0.001
ALP (U/l)	90.08 ± 29.01	84.46 ± 29.96	96.75 ± 26.33	<0.001
TBIL (U/l)	16.43 ± 7.11	16.33 ± 7.09	16.56 ± 7.14	0.302
UA (µmol/l)	300.71 ± 81.33	273.3 ± 69.66	333.3 ± 82.16	<0.001
UREA (µmol/l)	5.28 ± 1.51	5.12 ± 1.53	5.45 ± 1.45	<0.001
HP				0.004
No	1944 (47.64)	1102 (56.69)	842 (43.31)	
Yes	2137 (52.36)	1115 (52.18)	1022 (47.82)	

The data were present as mean ± SD or *n* (%). BMI, body mass index; TC, total cholesterol; TG, triglyceride; HDL, high-density lipoprotein; LDL, low-density lipoprotein; UA, uric acid; MS, metabolic syndrome; CR, creatinine.

### Association Between HP infection and NAFLD

Multivariable logistic regressions were conducted to examine the association of HP infection with NAFLD. As shown in Table 2, the OR and 95% CI of participants with HP infection for NAFLD were 1.20 (1.06–1.36) in the crude Model. After further adjusting for BMI, ALT, AST, AKP, TBIL, UR, and UREA, the OR and 95% CI of participants with HP infection for NAFLD were 1.27 (1.07–1.50) (Model 3).

**Table 2:** Association between HP infection and NAFLD.

	HP		*P*-value
	No	Yes	
Crude model	Ref	1.20 (1.06–1.36)	0.004
Model 1	Ref	1.17 (1.03–1.34)	0.014
Model 2	Ref	1.20 (1.05–1.38)	0.010
Model 3	Ref	1.27 (1.07–1.50)	0.006

Model 1: adjusted for sex and age; Model 2: adjusted for sex, age, education level, smoking, hypertension, diabetes, and dyslipidemia; Model 3: adjusted for sex, age, education level, smoking, hypertension, diabetes, dyslipidemia, BMI, ALT, AST, AKP, TBIL, UA, and UREA.

### Sensitivity Analysis in Different Groups

As shown in Table 3, stratified analyses were performed to explore the association between HP infection and NAFLD. Stratified analyses were made for age, sex and dyslipidemia. Participants were divided into three groups according to their age: <40, 40–59, and ≥60. Sex classifications were males and females, and dyslipidemia was indicated as yes or no. After adjusting for sex, age, smoking, education level, hypertension, diabetes, dyslipidemia, MS, BMI, ALT, AST, AKP, TBIL, UR and UREA, the fully adjusted OR and 95% CI for NAFLD were 1.22 (1.10–1.80) in females and 1.44 (1.18–1.75) in subjects with dyslipidemia. There were not significant increased OR for NAFLD when stratified by age.

**Table 3:** Relationship Between HP infection and NAFLD stratified by age, sex and Dyslipidemia.

	Crude model	Model 1	Model 2	Model 3
**Age (Years)**				
<40	1.11 (0.91–1.34)	1.07 (0.87–1.32)	1.09 (0.87–1.36)	1.21 (0.89–1.64)
40–59	1.22 (0.99–1.50)	1.24 (0.99–1.54)	1.26 (0.99–1.67)	1.26 (0.96–1.67)
≥60	1.24 (0.93–1.66)	1.30 (0.99–1.70)	1.29 (0.97–1.72)	1.36 (0.99–1.88)
**Sex**				
Male	1.16 (0.96–1.39)	1.15 (0.96–1.38)	1.13 (0.92–1.37)	1.22 (0.96–1.53)
Female	1.24 (1.04–1.48)	1.24 (1.03–1.49)	1.30 (1.06–1.58)	1.22 (1.10–1.80)
**Dyslipidemia**				
No	0.97 (0.75–1.26)	0.96 (0.74–1.26)	0.95 (0.72–1.25)	0.85 (0.60–1.20)
Yes	1.36 (1.16–1.58)	1.32 (1.13–1.55)	1.31 (1.12–1.54)	1.44 (1.18–1.75)

Model 1: adjusted for sex and age; Model 2: adjusted for sex, age, education level, smoking, hypertension, diabetes, and dyslipidemia; Model 3: adjusted for sex, age, education level, smoking, hypertension, diabetes, dyslipidemia, BMI, ALT, AST, AKP, TBIL, UA, and UREA.

## Discussion

The present study demonstrated that HP infection is independently associated with increased risk of NAFLD. The positive correlation was stronger in females and patients with dyslipidemia. There were no significant associations between HP infection and NAFLD in every age group.

Many clinical studies have been conducted to examine the relationship between HP infection and NAFLD. A systematic review and meta-analysis consisted of 38622 subjects identified a significantly increased risk of NAFLD in patients with HP infection^16^. Moreover, a cohort study found that the hazard ratio (HR) for NAFLD development in participants with HP infection was 1.21 (1.10–1.34)^19^. Whereas, a cross-sectional study performed in 13737 Japanese indicated that BMI, ALT and platelet count but not HP infection were significantly associated with NAFLD^17^. In addition, a retrospective cohort study performed in a 3663 Koreans revealed that HP infection was not a risk factor, but smoking was a significant risk factor for NAFLD^18^. Recently, a cross-sectional study carried out in South Chinese who underwent a healthy checkup program showed that NAFLD was significantly associated with HP infection in women but not in men; however, the association was not significant after adjusting for confounding factors including age, sex, BMI, blood pressure and lipid profiles^20^. The present study was performed in North Chinese and demostrated that HP infection is independently associated with increased risk of NAFLD, especially in females and patients with dyslipidemia. The inconsistent between the present study and the studies conducted in South Chinese, might caused by the regional variation (North China vs. South China), the population recruitment (community vs. heterogeneous subjects who underwent a healthy checkup program), and the confounding factors (we further adjusted education and smoking status). The inconsistent between the present study and the previous findings, might result from the ethnic or regional differences, which need further validated in case-control or longitudinal studies with good design, large sample size and multiple ethnic participants. Taken together, our study repeatedly validated that HP infection is independently associated with increased risk of NAFLD.

The pathogenesis that HP infection contribute to NAFLD is not well recognized. An animal study showed that HP infection contributed to the development of liver cirrhosis^21^. Moreover, HP infection was found to be significantly and independently associated with dyslipidemia^22^. Other studies found that serum triglycerides levels in HP positive groups were significantly higher than HP negative groups^23,24^. In this study, we also showed that the association between HP infection and NAFLD was statistically significant in the participants with dyslipidemia but not in those without dyslipidemia. HP infection has also been recognized to be the causal factor of insulin resistance (IR), chronic low-grade systemic inflammation, the inhibition white adipose tissue to release leptin and gastrointestinal flora dysbiota, which are the causal factors of NAFLD^25^. Therefore, HP infection might be of causal effect to the incidence of NAFLD, via the modification the serum lipid profile or metabolic pathway. Further understanding the pathogenic role of HP infection in NAFLD is important for devising new treatment strategies.

This study also had some limitations. Firstly, the ^13^C urea breath test is not the gold standard for HP detection. However, it is a highly sensitive, specific, and noninvasive diagnostic test for HP infection^26^. Secondly, NAFLD was not determined by liver biopsy. However, abdominal ultrasonography is thought to be fairly robust in detecting NAFLD^18^. In addition, due to the limitation in the study design (cross-sectional study), it is difficult to infer the causal effect relationship between HP infection and NAFLD. Therefore, further longitudinal cohort studies are needed to confirm the association between HP infection and NAFLD.

In conclusion, HP infection is associated with NAFLD, especially in females and patients with dyslipidemia. Considering that the high prevalence, unclear etiology and difficult treatment of NAFLD, confirming the association between HP infection and NAFLD will undoubtedly provide insights for novel prevention or treatment strategies for NAFLD.

## Methods

### Ethics statement

This study was performed according to the principles of the Helsinki Declaration and Good Clinical Practice (GCP) guidelines. The study protocol was approved by the Ethics Committee of the Staff Hospital of Jidong Oil Field of the Chinese National Petroleum Corporation (Tangshan, China). All of the subjects provided written informed consents.

### Subjects

The participants were recruited from the Caofeidian District, Tangshan City, China. A total of 6,656 participants agreed to be enrolled in the present study. Each participant was required to have a medical examination, provide blood samples, and complete a standard questionnaire during a face-to-face interview. In this study, participants who had undergone an abdominal ultrasound and a ^13^C urea breath test were included. Participants who were heavy drinkers (≥20 g/day for men and ≥10 g/day for women for more than a year), seropositive for hepatitis B virus surface antigen or had a history of cancer or stroke were excluded. Finally, 4,081 participants were included in this study (1887 men and 2194 women).

### Assessment of the presence of HP

Experienced examiners performed ^13^C urea breath tests (HG-IRIS13C Infrared Spectrometer, Beijing Richen-Force Science & Technology Co. Ltd., Beijing, China) to determine the HP infection status of the subjects, according to the manual instruction^27^.

### Assessment of NAFLD

Abdominal ultrasonography was performed by two well-trained examiners using a high-resolution B-mode topographic ultrasound system (ACUSON X300, Siemens, Germany). All of the examiners were blind to the health status and laboratory test results of the participants. NAFLD was diagnosed on the basis of characteristic sonographic features, including hepatorenal echo contrast, liver parenchymal brightness, deep beam attenuation, and vessel blurring^28^.

### Assessment of covariates

Trained examiners administered face-to-face interviews and collected information on biographical factors (age, sex, education), lifestyle factors [smoking status (No, Yes), drinking status (No, Yes)], education level, environmental exposure, and medical history^29^. Blood samples were collected after a 12-hour fast. Laboratory tests for fasting plasma glucose, glycated hemoglobin (Hb), triglyceride (TG), total cholesterol (TC), high-density lipoprotein (HDL), low-density lipoprotein (LDL), alanine aminotransferase (ALT), aspartate transaminase (AST), AST/ALT, alkaline phosphatase (ALP), total bilirubin (TBIL), urea (UREA), uric acid (UA), were performed at the central laboratory of the Staff Hospital of the Jidong Oil Field of the Chinese National Petroleum Corporation as described previously^29^. Body mass index (BMI) and blood pressures were measured as previous description, and the diagnosis of Hypertension, obesity, overweight, Diabetes mellitus (DM), MS can be referred to our previous studies^30,31^.

### Statistical analysis

Categorical variables were described as percentage (%) and compared by Chi-square tests. Normal distributed continuous variables were described as the mean ± standard deviation (SD) and compared with ANOVA or *t* test. the multivariable logistic regression models were used to calculate the odds ratio (OR) and 95% confidence interval (95% CI) for NAFLD. Adjustments were made for 15 variables (sex, age, smoking, education, hypertension, DM, dyslipidemia, MS, BMI, ALT, AST, ALP, TBIL, UA, and UREA) that were identified as potential confounders of the risk factors for NAFLD. Sensitivity analyses were used to estimate the association between HP infection and NAFLD, stratified by age, sex and dyslipidemia. Statistical analyses were performed using SAS software (Version 9.4, SAS Institute, Cary, NC, USA). All statistical tests were 2-sided, and significance levels were 0.05.
